# Supplementary material for: The Role of Intraventricular Hemorrhage in Traumatic Brain Injury: A Novel Scoring System
Source: J Clin Med. 2022 Apr 11;11(8):2127. doi: 10.3390/jcm11082127 (PMC9028147; doi:10.3390/jcm11082127)
Supplement: Supplementary file 1 [file jcm-11-02127-s001.zip › Supplementary Table S1.pdf]

Supplementary Table S1. Types of IVH grading scale and outcomes AUC (95% CI)

Outcomes / score	AUC (95% CI)	*P*	Cut-point	Sensitivity (95%CI)	Specifity (95%CI)
**In-hospital Mortality (*n* = 149)**					
The Graeb score	0.847 (0.779–0.901)	<0.001	>5	65.79 (48.6-80.4)	91.89 (85.2-96.2)
The LeRoux score	0.844 (0.775–0.898)	<0.001	>6	68.42 (51.3-82.5)	87.39 (79.7-92.9)
IVH score	0.828 (0.757–0.885)	<0.001	>10	68.42 (51.3-82.5)	88.29 (80.8-93.6)
Traumatic Graeb score	0.888 (0.826–0.934)	<0.001	>5	81.58 (65.7-92.3)	83.78 (75.6-90.1)
Traumatic LeRoux score	0.880 (0.817–0.927)	<0.001	>5	84.21 (68.7-94.0)	77.48 (68.6-84.9)
Traumatic IVH score	0.856 (0.789–0.908)	<0.001	>10	76.32 (59.8-88.6)	82.88 (74.6-89.4)
**Poor outcomes at discharge (mRS > 2)**					
The Graeb score*	0.821 (0.750–0.879)	<0.001	>2	75.29 (64.7-84.0)	76.56 (64.3-86.2)
The LeRoux score*	0.793 (0.718–0.854)	<0.001	>2	76.47 (66.0-85.0)	70.31 (57.6-81.1)
IVH score	0.813 (0.741–0.872)	<0.001	>5	85.88 (76.6-92.5)	59.38 (46.4-71.5)
Traumatic Graeb score	0.880 (0.817–0.928)	<0.001	>3	88.24 (79.4-94.2)	73.44 (60.9-83.7)
Traumatic LeRoux Score	0.852 (0.785–0.905)	<0.001	>3	88.24 (79.4-94.2)	68.75 (55.9-79.8)
Traumatic IVH score	0.861 (0.795–0.912)	<0.001	>7	80.00 (69.9-87.9)	81.25 (69.5-89.9)

Abbreviations: IVH, intraventricular hemorrhage; AUC: area under the curve; CI: confidence interval; mRS, modified Rankin Scale

*: The Graeb score was significanly more accurate than the LeRoux score in predicting poor outcomes
