# Supplementary material for: The Role of Intraventricular Hemorrhage in Traumatic Brain Injury: A Novel Scoring System
Source: J Clin Med. 2022 Apr 11;11(8):2127. doi: 10.3390/jcm11082127 (PMC9028147; doi:10.3390/jcm11082127)
Supplement: Supplementary file 1 [file jcm-11-02127-s001.zip › Supplementary Table S2.pdf]

Supplementary Table S2. ΔAUC values of different models in predicting outcomes

Outcomes / score	ΔAUC, % (95% CI)	*P*
In-hospital Mortality		
**Traumatic Graeb score†**	Reference	-
The Graeb score*	4.09 (0.82–7.36)	0.014
The LeRoux score*	5.44 (0.63–8.19)	0.022
IVH score*	6.03 (0.31–11.80)	0.039
**Traumatic LeRoux score**	Reference	-
The Graeb score	3.27 (-0.43–6.97)	0.083
The LeRoux score*	3.59 (0.74–6.44)	0.014
IVH score	5.22 (-1.45–11.90)	0.125
**Traumatic IVH score**	Reference	-
The Graeb score	0.89 (-4.03–5.81)	0.723
The LeRoux score	1.21 (-5.21–7.63)	0.712
IVH score*	2.83 (0.69–4.98)	0.001
Poor outcomes at discharge (mRS > 2)		
**Traumatic Graeb score†**	Reference	-
The Graeb score*	5.97 (2.84–9.11)	<0.001
The LeRoux score*	8.84 (4.68–13.00)	<0.0001
IVH score*	6.71 (1.65–11.80)	0.009
**Traumatic LeRoux score**	Reference	-
The Graeb score	3.12 (-0.27–6.52)	0.071
The LeRoux score*	5.99 (3.13–8.85)	<0.001
IVH score	3.86 (-1.81–9.53)	0.182
**Traumatic IVH score**	Reference	-
The Graeb score	4.06 (-0.66–8.79)	0.092
The LeRoux score*	6.93 (1.16–12.70)	0.019
IVH score*	4.80 (2.62–6.98)	<0.001

Abbreviations: IVH, intraventricular hemorrhage; AUC: area under the curve; CI: confidence interval; mRS, modified Rankin Scale

†: The only score that surpassed all three existing IVH grading scales in predicting outcomes.

***:** ΔAUC that was statistically significant.
