# Supplementary material for: The Role of Intraventricular Hemorrhage in Traumatic Brain Injury: A Novel Scoring System
Source: J Clin Med. 2022 Apr 11;11(8):2127. doi: 10.3390/jcm11082127 (PMC9028147; doi:10.3390/jcm11082127)
Supplement: Supplementary file 1 [file jcm-11-02127-s001.zip › Supplementary Table S3.pdf]

Supplementary Table S3. Traumatic Graeb score and outcomes of IVH in different ventricles AUC (95% CI)

Outcomes / score	AUC (95% CI)	*P*	Cut-point	Sensitivity (95%CI)	Specifity (95%CI)
In-hospital Mortality					
All (*n* = 149)	0.888 (0.826–0.934)	<0.001	>5	81.58 (65.7-92.3)	83.78 (75.6-90.1)
Lateral ventricle (*n* = 136)	0.888 (0.822–0.935)	<0.001	>5	83.33 (67.2-93.6)	82.18 (73.3-89.1)
3rd ventricle (*n* = 51)	0.865 (0.740–0.944)	<0.001	>7	84.00 (63.9-95.5)	73.08 (52.2-88.4)
4th ventricle (*n* = 59)	0.898 (0.791–0.961)	<0.001	>7	84.00 (63.9.-95.5)	79.41 (62.1-91.3)
Poor outcomes at discharge (mRS > 2)					
All (*n* = 149)	0.880 (0.817–0.928)	<0.001	>3	88.24 (79.4-94.2)	73.44 (60.9-83.7)
Lateral ventricle (*n* = 136)	0.865 (0.796–0.917)	<0.001	>3	87.80 (78.7-94.0)	69.09 (55.2-80.9)
3rd ventricle (*n* = 51)	0.857 (0.730–0.939)	<0.001	>7	67.50 (50.9-81.4)	90.91 (58.7-99.8)
4th ventricle (*n* = 59)	0.891 (0.783–0.957)	<0.001	>6	71.43 (55.4.-84.3)	88.24 (63.6-98.5)

Abbreviations: IVH, intraventricular hemorrhage; AUC: area under the curve; CI: confidence interval; mRS: modified Rankin Scale
