# Supplementary material for: The Role of Intraventricular Hemorrhage in Traumatic Brain Injury: A Novel Scoring System
Source: J Clin Med. 2022 Apr 11;11(8):2127. doi: 10.3390/jcm11082127 (PMC9028147; doi:10.3390/jcm11082127)
Supplement: Supplementary file 1 [file jcm-11-02127-s001.zip › Supplementary Table S4.pdf]

Supplementary Table S4. Poor functional outcomes in different Traumatic Graeb Score

Traumatic Graeb Score	N	Poor functional outcomes (%)
1	14	7.1
2	27	7.4
3	25	52
4	27	66.7
5	11	90.9
6	11	72.7
7	4	100
8	9	100
9	7	85.7
10	8	100
11	3	100
12	1	100
13	1	100
14	1	100
