# Supplementary figures and images for: The Role of Intraventricular Hemorrhage in Traumatic Brain Injury: A Novel Scoring System
Source: J Clin Med. 2022 Apr 11;11(8):2127. doi: 10.3390/jcm11082127 (PMC9028147; doi:10.3390/jcm11082127)

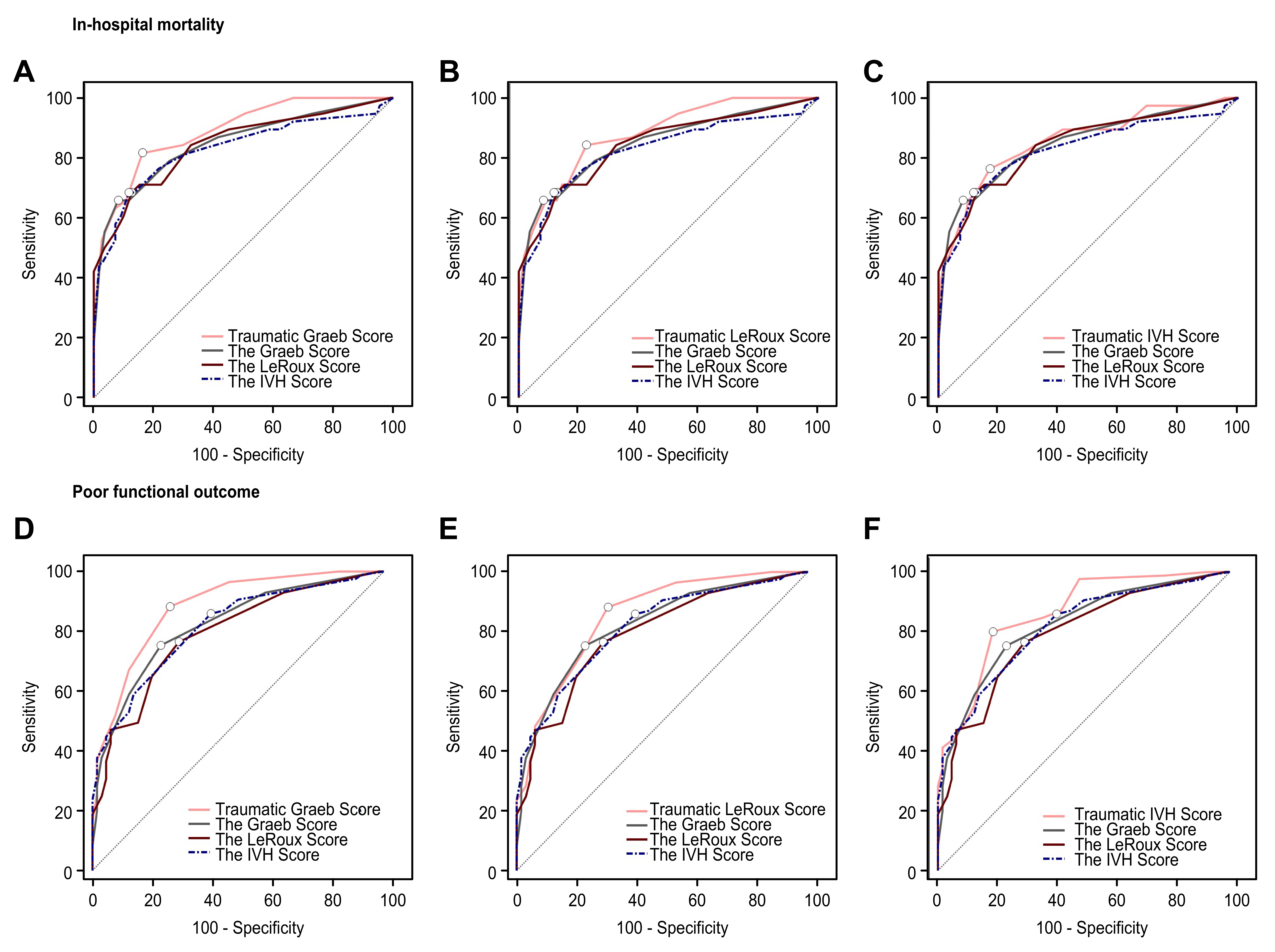

Supplement: Supplementary file 1 [file jcm-11-02127-s001.zip › Supplementary Figure S1.tif]
